# Postoperative Pain Management With Ketorolac, Acetaminophen, and Gabapentin in Femoral Shaft Fractures: A Prospective Cohort Study

**DOI:** 10.5435/JAAOSGlobal-D-25-00027

**Published:** 2025-05-08

**Authors:** Hans Hess Arcelay, José I. Acosta Julbe, Alexandra M. Claudio-Marcano, Francis X. Cedeño Rodriguez, Edgardo Martinez, Ruben Tresgallo, Norman Ramirez Lluch, Luis Lojo-Sojo

**Affiliations:** From the Department of Orthopedic Surgery, University of Puerto, Río Piedras, Puerto Rico (Dr. Hess Arcelay, Dr. Acosta Julbe, Dr. Claudio-Marcano, Dr. Martinez, and Dr. Lojo-Sojo); the School of Medicine, University of Puerto Rico, San Juan, Puerto Rico (Mr. Cedeño Rodriguez); the Division of Arthroplasty, Department of Orthopaedic Surgery, Brigham and Women's Hospital, Boston, MA (Dr. Tresgallo); the Department of Orthopaedic Surgery, Mayagüez Medical Center, Mayagüez, Puerto Rico (Dr. Ramirez Lluch); and the School of Medicine, Ponce Health Sciences University, Ponce, Puerto Rico (Dr. Ramirez Lluch).

## Abstract

**Background:**

Postoperative pain management in orthopaedic trauma surgery is critical. Amid the opioid crisis, nonopioid analgesia offers a promising alternative. We aim to evaluate the feasibility of a multimodal regimen, including intravenous ketorolac, oral acetaminophen, and oral gabapentin, in managing postoperative pain after femur shaft fractures treated with intramedullary nailing.

**Methods:**

We conducted a prospective cohort study to evaluate the postoperative pain of patients with isolated femur shaft fractures treated with intramedullary nailing. The experimental group consisted of 29 patients, while the control group consisted of 42 patients who received standard opioid-based pain management. The outcomes measured were the Visual Analog Scale (VAS) scores and the morphine milligram equivalents every 12 hours until 48 hours postoperatively.

**Results:**

Patient demographics were comparable between the experimental and control groups, with a significant age difference (31 vs. 40; *P* = 0.002) but not in sex, body mass index, smoking status, or diabetes prevalence. We observed no significant differences in VAS scores at 12, 24, 36, and 48 hours after surgery. In addition, there was a marked reduction in opioid usage in the experimental group, with *P*-values < 0.001 at each time point.

**Conclusion:**

The study concludes that the coadministration of ketorolac, acetaminophen, and gabapentin is an effective opioid-sparing alternative for postoperative pain in isolated femur shaft fractures. Future research could benefit from a more diverse patient population in orthopaedic trauma to further validate our results and explore long-term outcomes.

**Level of Evidence:**

Prospective Cohort Study; Level 2.

Effective postoperative pain management is critical in orthopaedic surgery, especially for femoral shaft fractures treated with intramedullary nailing (IMN). The ongoing opioid crisis highlights the need for nonopioid alternatives that provide effective pain relief without associated risks.^1^ Standard approaches often rely on opioid analgesics, which, despite their effectiveness, have been increasingly scrutinized because of the risk of addiction and adverse side effects contributing to the ongoing opioid crisis.^1^ This concern has prompted a search for nonopioid alternatives that can provide effective pain relief without the associated risks.

Literature suggests that multimodal pain management strategies, which employ the use of various nonopioid analgesics, can potentially reduce the reliance on opioids without compromising pain control.^2^ Among the alternatives, intravenous (IV) ketorolac and per-oral (PO) acetaminophen have emerged as viable options, with studies demonstrating their potential to reduce the need for opioids for pain management, thereby minimizing associated risks.^3,4^ In addition, a meta-analysis conducted by Yu et al.^5^ showed that oral gabapentin was efficacious in the reduction of postoperative pain and narcotic requirements after lumbar spinal surgery. Despite promising results, the synergy of IV ketorolac, oral acetaminophen, and oral gabapentin has not been thoroughly examined in managing femur fractures.

We aim to assess the effect of the combination of IV ketorolac, oral acetaminophen, and oral gabapentin in comparison with an opioid-based regimen on pain control and opioid consumption during the first 48 hours after IMN of isolated femur shaft fractures. We used the Visual Analog Scale (VAS) scores and morphine milligram equivalents (MMEs) as our primary outcome measures. We hypothesize that the administration of combined IV ketorolac, oral acetaminophen, and oral gabapentin to patients undergoing IMN for isolated closed femur shaft fractures will decrease opioid consumption while achieving pain control comparable with that of standard opioid-based methods.

## Methods

### Patients

We screened consecutive patients with closed femur shaft fractures (Arbeitsgemeinschaft für Osteosynthesefragen/Orthopaedic Trauma Association 32) undergoing IMN from April 2021 to October 2022. Eligibility criteria included adult patients aged between 21 and 65 undergoing IMN to treat closed femur shaft fractures. Exclusion criteria encompassed recent opioid use, nonsteroidal anti-inflammatory hypersensitivity, renal/cardiac/hepatic impairment, gastrointestinal bleeding history, coagulopathy, substance abuse diagnosis, open fractures, polytrauma, pregnancy, and inability to consent. Participants were excluded from the final analysis if they could not receive the protocol or had incomplete data.

### Study Design

This prospective cohort study evaluated a nonopioid pain management protocol against a standard opioid-based regimen in patients undergoing IMN for closed femur shaft fractures. Patients were assigned to one of the two cohorts based on the pain management protocol chosen by the attending surgeon. Cohort assignments reflected clinical practice preferences without randomization, ensuring that the study observed real-world treatment scenarios.

Data collection was conducted prospectively by a staff physician blinded to the treatment protocol assigned. This research assistant was not involved in patient care or data collection to minimize bias. All procedures were conducted at a Level I trauma center, and patients were admitted to the same orthopaedic ward.

### Pain Management Protocols

Patients were assigned to an experimental (nonopioid-based) group and a control (opioid-based) group. Before surgery, the experimental group received 30 mg of IV ketorolac. After surgery, the experimental group received 30 mg of IV ketorolac every six hours, 1 g of acetaminophen PO every 6 hours, and 300 mg of gabapentin PO every 12 hours. The control group received morphine (0.1 mg/kg IV) and oxycodone with acetaminophen (2.5/325 mg) PO every 6 hours after surgery, starting at the postanesthesia care unit. Opioid rescue medication was available as needed for both cohorts.

### Primary Outcome Measures

Pain scores were evaluated using the VAS score and assessed at 12-hour intervals from 12 to 48 hours postoperatively. The VAS score is a validated questionnaire for postsurgical pain evaluation in which scores are recorded by making a handwritten mark on a 10-cm line representing a continuum between “no pain” and “worst pain.”^6^ Opioid consumption was quantified using the MMEs and measured 12, 24, 36, and 48 hours after surgery. While trends were observed at all time points, we designated 24 hours postoperatively as the primary time point for statistical comparison between the intervention and control groups. This time point was chosen because of its clinical relevance in the acute recovery phase.

### Statistical Analysis

Descriptive statistics were conducted using frequency and percentages for categorical variables and medians with interquartile ranges for quantitative data. The Student *t*-test assessed continuous variables' means, with a *P*-value ≤ 0.05 indicating statistical significance. Because this was a pilot study, no formal power analysis was conducted. Instead, we aimed to estimate feasibility and effect size for future trials. Statistical analyses were performed using Microsoft Excel and Statistical Package for the Social Sciences statistical software.

## Results

### Demographics

We analyzed two cohorts: the experimental group, with 29 patients receiving nonopioid pain management, and the control group, with 52 patients receiving standard opioid-based pain management after IMN of isolated femur shaft fractures. Ten patients from the control group were lost to follow-up (i.e., abandoned the ward before data collection or incomplete data collection). Patient demographics were comparable between the experimental and control groups, with a significant age difference (31 vs. 40; *P* = 0.002) but not in sex, body mass index, smoking status, or diabetes prevalence (Table 1).

**Table 1:** Patient Demographics

Variables	Experimental Group (n = 29 Patients)	Control Group (n = 42 Patients)	*P*
Age (yrs, median, IQR)	31 (23 to 42)	40 (27 to 60)	**0.002**
Sex			0.337
Female (n, %)	6 (21%)	13 (31%)	
Male (n, %)	23 (79%)	29 (69%)	
BMI (kg/m^2^, mean, SD)	27.1 (SD 7.1)	26.7 (SD 6.2)	0.799
Smoker (n, %)			0.337
No	23 (79%)	29 (69%)	
Yes	6 (21%)	13 (31%)	
Diabetes (n, %)			0.319
No	26 (90%)	34 (81%)	
Yes	3 (10%)	8 (19%)	

IQR = interquartile range

Bold entries = statistical significance, as the *p*-value is less than 0.05.

### Pain Outcomes

VAS pain scores were collected at 12-hour intervals over a 48-hour follow-up period. VAS scores at 12, 24, 36, and 48 hours after treatment revealed no significant differences between both groups, ensuring that the nonopioid regimen provided adequate pain control (Table 2).

**Table 2:** Visual Analog Scale (VAS) Pain Scores Over the 48-Hour Follow-Up Period

Variables	Experimental Group (n = 29 Patients)	Control Group (n = 42 Patients)	*P*
VAS 12 hr	7.3 (SD 2.5)	7.2 (SD 2.0)	0.445
VAS 24 hr	5.4 (SD 2.6)	5.1 (SD 2.4)	0.300
VAS 36 hr	5.3 (SD 1.8)	5.6 (SD 2.3)	0.359
VAS 48 hr	4.0 (SD 1.6)	3.4 (SD 2.2)	0.152

### Opioid Consumption

The MMEs consumed in the experimental group were markedly lower than in the control group at all measured time intervals. The median MME consumption in the experimental group was consistently less than half that of the control group, with statistically significant reductions (*P* < 0.001) observed at 12, 24, 36, and 48 hours (Table 3). One patient from the experimental group required a single rescue dose.

**Table 3:** Morphine Milligram Equivalents Over the 48-Hour Follow-Up Period

Variables	Experimental Group (n = 29 Patients)	Control Group (n = 42 Patients)	*P*
MME 12 hr	2.9 (SD 4.5)	12.1 (SD 11.6)	**<0.001**
MME 24 hr	10.4 (SD 13.6)	28.8 (SD 20.6)	**<0.001**
MME 36 hr	15.7 (SD 15.9)	39.6 (SD 29.1)	**<0.001**
MME 48 hr	20.1 (SD 21.0)	48.0 (SD 35.6)	**<0.001**

MME = morphine milligram equivalent

Bold entries = statistical significance, as the *p*-value is less than 0.05.

## Discussion

Effective postoperative pain management in orthopaedic trauma patients, particularly after femoral shaft fractures managed with IMN, remains a notable challenge. While opioids have historically formed the foundation for managing acute postoperative pain due to their effectiveness, their potential for long-term dependence and numerous side effects underscores the need for alternative strategies.^1^ Recent efforts have aimed to provide evidence supporting opioid-sparing modalities in orthopaedic surgery.^7-9^ Our findings offer preliminary evidence that a multimodal, nonopioid approach, incorporating ketorolac, acetaminophen, and gabapentin, can effectively manage postoperative pain while reducing opioid consumption after isolated femoral shaft fractures managed with IMN.

The VAS score is the most used variable to measure postoperative pain management, which provides a quantifiable measure of the patient's pain experience.^6^ We observed that at 12 hours after treatment, the experimental group reported VAS scores with a mean of 7.3, closely paralleling the control group's mean of 7.2. This equivalence in pain perception continued consistently throughout the 48-hour assessment period, signifying that the nonopioid pain management strategy provided pain control comparable with the opioid regimen. The uniformity in VAS scores reinforces the effectiveness of the nonopioid approach in maintaining equivalent pain relief to standard opioid-based methods. ^7^ Another method of measuring the efficacy of an opioid-sparing pain management protocol is the MMEs. In addition, the notable reduction in opioid consumption within the experimental group, less than half the median MMEs compared with the control group across all time intervals, supports the increasing preference for multimodal pain management strategies. These strategies prioritize minimizing opioid usage without compromising pain control, thereby supporting patient recovery.^4,8,9^

The risks associated with opioid use, including respiratory depression, constipation, nausea, vomiting, physical dependence, and the potential for misuse and addiction, have led to a re-evaluation of their role in pain management. Moreover, the widespread use of opioids has contributed to a public health crisis, with increasing rates of opioid-related overdoses and deaths.^1^ Research has shown that postoperative opioid prescriptions can be a gateway to prolonged use, highlighting the necessity for nonopioid options in postsurgical care. For example, Goesling et al^10^ demonstrated that a notable portion of patients continue to use opioids well beyond the acute recovery phase, leading to increased risks of chronic use and dependence. Furthermore, Brummett et al linked postoperative opioid-prescribing practices to the likelihood of persistent opioid use, underlining the importance of considering alternative pain management strategies to mitigate these risks.^10,11^

Considering these issues, our study's results contribute to the growing consensus that multimodal analgesia can be both adequate and preferable to opioid-centric approaches.^3,8^ Singla et al^2^ demonstrated a notable reduction in pain and morphine use after the use of IV ibuprofen for postoperative orthopaedic patients, supporting our findings. Liang et al^3^ found that IV acetaminophen after total joint arthroplasty produced a decrease in pain scores and opioid consumption. Furthermore, a systematic review by Ladich et al^12^ found oral gabapentin to be both statistically and clinically significant in reducing postoperative pain and opioid consumption during the immediate postoperative period, further substantiating the role of nonopioid analgesics in postoperative pain management.

We acknowledge several limitations within our study. First, the follow-up period was designed to assess immediate postoperative outcomes and was not extended to evaluate long-term effects, such as bone healing and functional outcomes. Because this cohort study was not randomized, the observed differences in baseline characteristics reflect real-world variability rather than controlled allocation. While this provides valuable insights, it also introduces potential confounding variables that could influence the results. In addition, the small sample size limits the generalizability of our findings. Although appropriate for a pilot study, future research should include larger, more diverse cohorts to validate these results further and explore long-term outcomes. Finally, because the VAS score for pain is a subjective measurement, there is an inherent probability of underperception or overperception of pain. Future studies should address these limitations and incorporate additional objective measures for pain assessment.

The ongoing opioid crisis has prompted a re-evaluation of pain management strategies in orthopaedic surgery. Our study supports the adoption of IV ketorolac, oral acetaminophen, and oral gabapentin as a viable alternative to opioids for postoperative pain management in orthopaedic surgery, specifically IMN of isolated femur shaft fractures. With orthopaedic surgeons being among the highest prescribers of opioids in the United States, the shift toward multimodal, nonopioid analgesia, as demonstrated in our study, is a crucial step in addressing this public health issue.^13^
